# Human IgG Antibody Response to *Aedes* Nterm-34kDa Salivary Peptide, an Epidemiological Tool to Assess Vector Control in Chikungunya and Dengue Transmission Area

**DOI:** 10.1371/journal.pntd.0005109

**Published:** 2016-12-01

**Authors:** Emmanuel Elanga Ndille, Souleymane Doucoure, Anne Poinsignon, François Mouchet, Sylvie Cornelie, Eric D’Ortenzio, Jean Sébastien DeHecq, Franck Remoue

**Affiliations:** 1 Institut de Recherche pour le Développement (IRD), Maladies Infectieuses et Vecteurs, Ecologie, Génétique, Evolution et Contrôle (MIVEGEC), Montpellier, FRANCE; 2 Institut de Veille Sanitaire, Cire Océan Indien, Saint-Denis, La Réunion; 3 Agence Régionale de Santé, Océan Indien, Saint Denis, La Réunion; Centers for Disease Control and Prevention, Puerto Rico, UNITED STATES

## Abstract

**Background:**

Arboviral diseases are an important public health concerns. Vector control remains the sole strategy to fight against these diseases. Because of the important limits of methods currently used to assess human exposure to *Aedes* mosquito bites, much effort is being devoted to develop new indicators. Recent studies have reported that human antibody (Ab) responses to *Aedes aegypti* Nterm-34kDa salivary peptide represent a promising biomarker tool to evaluate the human-*Aedes* contact. The present study aims investigate whether such biomarker could be used for assessing the efficacy of vector control against *Aedes*.

**Methodology/Principal findings:**

Specific human IgG response to the Nterm-34kDa peptide was assessed from 102 individuals living in urban area of Saint-Denis at La Reunion Island, Indian Ocean, before and after the implementation of vector control against *Aedes* mosquitoes. IgG response decreased after 2 weeks (P < 0.0001), and remained low for 4 weeks post-intervention (P = 0.0002). The specific IgG decrease was associated with the decline of *Aedes* mosquito density, as estimated by entomological parameters and closely correlated to vector control implementation and was not associated with the use of individual protection, daily commuting outside of the house, sex and age. Our findings indicate a probable short-term decrease of human exposure to *Aedes* bites just after vector control implementation.

**Conclusion/Significance:**

Results provided in the present study indicate that IgG Ab response to *Aedes aegypti* Nterm-34kDa salivary peptide could be a relevant short-time indicator for evaluating the efficacy of vector control interventions against *Aedes* species.

## Author Summary

In absence of effective treatment and vaccine, vector control is the main strategy against arboviral diseases such as dengue, Zika and chikungunya. Given the limitation of entomologic tool currently used, news tools are urgently needed to assess the efficacy of vector control against arboviral diseases. The present study aimed to investigate whether human IgG antibody specific response to only one *Aedes* salivary peptide could be useful for assessing the efficacy of vector control against arboviral diseases. For this purpose, IgG response to Nterm-34kDa peptide was assessed from 102 individuals living in urban area at La Reunion Island, Indian Ocean, before and after the implementation of vector control against *Aedes albopictus* mosquito species. A significant decrease of this specific IgG level was noticed after vector control implementation. The decrease was associated to the decline in *Aedes* mosquito density estimated by entomological parameters, such as adult mosquito density, House and Breteau indices. The results of the present study indicated that human IgG response to the *Aedes* Nterm-34kDa salivary peptide could be a useful tool to evaluate the efficacy of vector control strategies against arboviruses.

## Introduction

Chikungunya and dengue fevers are diseases caused by chikungunya (CHIKV) and dengue (DENV) viruses, respectively. These viruses are transmitted to the human host by the bite of an infected *Aedes* mosquito, especially *Aedes aegypti* and *Aedes albopictus* mosquitoes [1,2]. During the past three decades, the range of the mosquito vectors has increased and dengue and chikungunya have become endemic in areas where they previously were not creating major public health problems in tropical and subtropical regions [1]. Currently, no specific therapeutic drugs or commercial vaccine are available and vector control remains the sole method for reducing transmission. Vector control strategies commonly used are based on: i) reduction of larval habitats by physical elimination of water-holding container and/or using larvicides and ii) control of adult mosquitoes by insecticide spraying. However, some recent techniques could be also effective *Aedes* mosquito control strategies such as: i) lethal ovitraps used for killing eggs, larvae, and female mosquitoes when they alight to oviposit, ii) transgene system such as RIDL RIDL, i.e. “Release of Insects carrying a Dominant Lethal which induce repressible female-specific lethality, iii) the use of Wolbachia-induced cytoplasmic incompatibility which can reduce mosquito life span and reproduction. The successful control of CHIKV and DENV transmission remains then linked to the efficacy of such anti-vector strategies.

The evaluation of vector control against CHIKV and DENV transmission, and other arboviruses such as Zika, is based on entomological methods, such as the identification and numbering of larval habitats, the collection of adult mosquitoes (by traps, pyrethrum spray or human lading catches) [3]. The indices of Breteau, Adult Productivity, House and Adult density are the most common indicators for evaluating the abundance of *Aedes* population [4]. Unfortunately, these indicators present numerous limitations regarding large-scale follow-up. The identification of larval habitats is very labor-time consuming. Indices based on *Aedes* immature stages are a poor proxy for measuring adult abundance and are not efficient for assessing transmission risk [4]. Estimation of adult mosquitoes abundance is most appropriate to assess transmission risk [4], but adults collection is fastidious and ethical concerns related to human lading catches may arise. In addition, these methods are mainly applicable at the community level and are not applied for evaluating the heterogeneity of the individual exposure to *Aedes* bites

Much effort is needed to develop new, sensitive and complementary indicators for measuring individual exposure to *Aedes* bites and efficacy of vector control, and are highlighted by the recent Zika virus epidemic. The measure of human antibody (Ab) response to *Aedes* salivary proteins represents a novel approach. Previous studies have shown that bioactive molecules in arthropod saliva, injected in human skin during the vector bites, could induce host immune reactions [5–7]. Recent studies have demonstrated the usefulness of anti-saliva Ab response for measuring exposure of humans to arthropods bites, such as, ticks [8] sand fly [9,10], *Glossina* [11] and mosquitoes [12–19]. Additionally, human Ab IgG response to whole saliva was identified as pertinent tool for evaluating efficacy of vector control against *Anopheles* [20,21], *Phlebotomus* [22] and *Aedes albopictus* [23]. However, the use of whole saliva is not an ideal indicator, because of: i) potential cross-reactivity with other vectors; ii) weak stability / fast degradation of proteins in wholes saliva and iii) poorly reproducible batches produced for large-scale studies. Optimization of this tool would be the identification of specific and antigenic salivary proteins and/or peptides. For example, only one gSG6-P1 salivary peptide, derived from gSG6 salivary protein, has been validated as specific biomarker of human exposure to *An*. *gambiae* and *An*. *funestus* bites [17,24–27], and has been used to evaluate the efficacy of insecticide treated nets against malaria transmission [28]. In regard to *Aedes* species, the 34kDa salivary protein appears to be antigenic and specific to *Aedes* genus [29–31]. One peptide (the Nterm-34kDa) from this protein in *Aedes aegypti* saliva has been recently validated, by several studies, as appropriate candidate biomarker of specific exposure to *Aedes* bites [32,33].

The present study investigated whether individuals from La Reunion Island (Indian Ocean), who are exposed to *Aedes albopictus* and not to *Aedes aegypti* species bites [34], presented specific Ab responses to *Aedes aegypti* Nterm-34kDa salivary peptide, and whether the level of this specific IgG response could be influenced by the implementation of vector control strategies against *Aedes* mosquito species. For this purpose, human IgG responses to the Nterm-34kDa peptide were measured in individuals, before and after vector control implementation (VCI).

## Materials and methods

### Ethics statement

This study followed ethical principles recommended by the Edinburgh revision of the Helsinki Declaration. The protocol was approved by a French Ethics Committee (the Sud-*Ouest*, *Outre Mer* Ethics Committee; 25^th^ February of 2009) and authorized by French Drug Agency (AFFSAPS, French Ministry of Health; 12^th^ January of 2009). Written informed consent was obtained from all subjects included in the study.

### Study site

The study was conducted in two urban districts of Saint-Denis, the largest city in La Reunion Island, situated in the Indian Ocean (21,8160 S; 55,8310 E), (23). During the massive chikungunya outbreak that occurred on 2005, about 36% of the inhabitants of this island were infected [35]. In 2009 and 2010, moderate outbreaks of chikungunya were also reported. Recently, autochthonous cases of DENV infection have been reported [36].

### Study design and population

A longitudinal study was carried out during the peak of *Ae*. *albopictus* abundance, from the 2nd of May to 9th of July 2010. Overall, 75 households and 102 individuals aged from 18 to 65 years, were randomly included by a “door to door” approach and according to the agreement of studied population to participate to the study. Each household was visited four times: before (T0) VCI and 15 (T0+15), 30 (T0+30), 45 (T0+45) days after VCI. The vector control implemented (VCI) was performed by the vector control unit of the Agence Régionale de la Santé (ARS) few days after the T0 visit and included: i) physical elimination of *Aedes* positive breeding sites combined with ii) spatial spraying of deltamethrin insecticide at 1g/ha concentration, twice two days apart, as previously described [23]. At each visit, artificial *Ae*. *albopictus* aquatic habitat were also physically eliminated by ARS team during each visit after VCI. A dried blood spot was collected from every individual at each visit (for immunological analysis). Standardized questionnaires were individually administered to collect information about individual protection against mosquito bites (use of bednets, mosquito repellent, mosquito coils, daily commuting out homes; i.e: getting out of the house every day for a professional activity). Dried blood spots (n = 10; “not exposed individuals”) were also collected during winter (February) from people who had not been out of France during the last four months before blood sampling, to serve as non-exposed control.

### Entomological data collection

The densities of *Aedes albopictus* adult mosquito were monitored every two days using four (two for each district) Mosquito Magnet® traps baited with CO_2_ and octenol. At each visit, all *Ae*. *albopictus* breeding sites were then physically eliminated. During the visits, larval indices were also calculated: i) House index (HI): percentage of houses infested with *Aedes* larvae and/or pupae and ii) Breteau index (BI): number of positive containers per 100 houses inspected.

### Nterm-34kDa salivary peptide

The Nterm-34 kDa salivary peptide has been selected as previously described [32] and synthesized and purified (>95%) by Genepep SA (St-Jean de Vedas, France). The peptides were shipped in lyophilized form and then suspended milliQ water and stored in aliquots at -20°C.

### Evaluation of human IgG antibody levels

Enzyme-linked immunosorbent assay (ELISA) was performed as previously described [32]. Briefly, the peptide (20μg/mL in 100 μl of Phosphate Buffer Saline, i;e. PBS) was coated for 150 minutes at 37°C into Maxisorp plates (Nunc, Roskilde, Denmark). Plates were blocked by Protein-Free Blocking-Buffer (Pierce, Thermo Scientific, France). Each eluate was incubated in triplicate at 4°C overnight at 1/20 dilution in PBS-Tween 1%. Mouse biotinylated Ab to human IgG (BD Biosciences, San Diego, CA) was incubated at a 1/1000 dilution in PBS-Tween 1% and peroxidase-conjugated streptavidin (GE Healthcare, Orsay, France) was added (1/1000 dilution in PBS-Tween 1%). Colorimetric development was carried out using 2, 2’-azino-bis (3-ethylbenzthiazoline 6-sulfonic acid) diammonium (ABTS; Thermo Scientific, France) and absorbance (OD) was measured at 405 nm. Individual results were expressed as the ΔOD value calculated according to the formula ΔOD = ODx − ODn, where ODx represented the mean of individual OD values in the two wells containing antigen and ODn the OD value in well without antigen. A subject was considered as an “immune responder” if ΔOD was higher than the cut-off (Cut-off = mean (ΔOD_unexposed_) + 3SD = 0.181) calculated from specific IgG level in negative “not exposed” controls (n = 10).

### Statistical analysis

After verifying that ΔOD values were not normally distributed, non-parametric tests were performed using GraphPad Prism5 software (San Diego, CA) to compare the ΔOD. The Mann–Whitney test was used for comparison of Ab levels of two independent groups and the Wilcoxon matched-pairs test was used for comparison of two paired groups. The non-parametric Kruskal–Wallis test was used for comparison of more than two groups and the Pearson’s Chi-squared test was used to compare two proportions. Moreover, a linear mixed effect regression (with individual as random effect) using R-software with the ‘nlme’ package, was performed for multivariate analysis of the specific IgG Ab response. All differences were considered significant at P < 0.05.

## Results

### IgG Ab response against Ae. aegypti Nterm-34kDa salivary peptide in individuals exposed to Ae. albopictus bites

**Fig 1:**
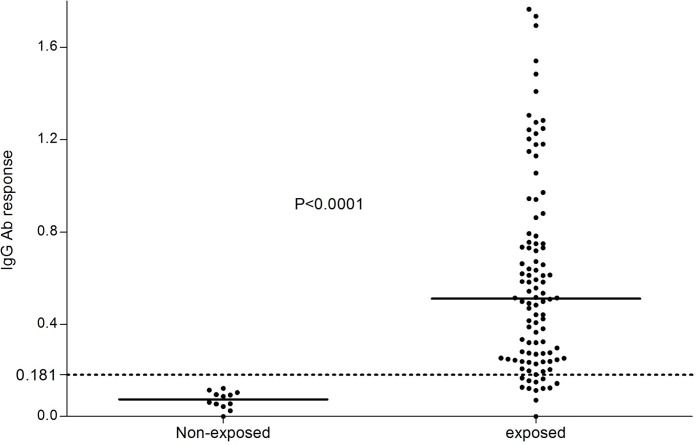
IgG Ab response to *Ae*. *aegypti* Nterm-34kDa salivary peptide in individuals exposed to *Ae*. *albopictus* bites at La Reunion Island and in non-exposed individuals. Black points indicate individual IgG response (ΔOD) and bars represent the median value in each group. Dotted line represents the cut-off of specific Ab response (ΔOD>0.181) and p-value was calculated using the Mann-Whitney U test.

The usefulness of measuring a human antibody response against *Ae*. *albopictus* using the Nterm-34kDa peptide was validated in individuals that were exposed to the bites of *Ae*. *albopictus* and in individuals that were not exposed (P < 0.0001, Mann-Whitney, Fig 1). Overall, 88.23% (90/102) of Reunionian individuals presented specific IgG responses which were higher than the cut-off. The median value (ΔOD = 0.510) of the specific IgG response from exposed individuals was 3 fold higher than the cut-off (ΔOD = 0.181). These results indicated the existence of high specific Ab response to *Ae*. *aegypti* Nterm-34kDa salivary peptide in human populations exclusively exposed to *Ae*. *albopictus* bites.

### Evaluation of IgG response to Nterm-34kDa salivary peptide before and after vector control implementation (VCI)

**Fig 2:**
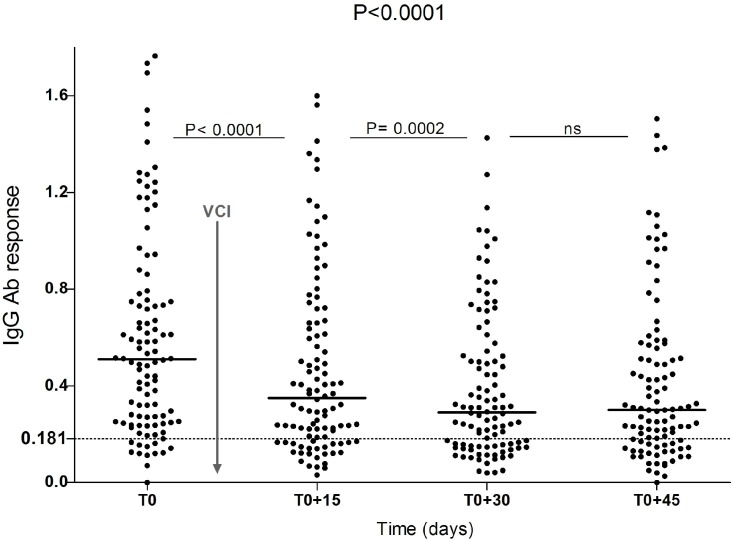
IgG Ab response to Nterm-34kDa salivary peptide from individuals exposed to *Ae*. *albopictus* bites before and after vector control implementation. Individual IgG Ab response (ΔOD) is presented just before (T0) and then 15, 30 and 45 days after vector control implementation. Bars indicated the median value in the population at each time point and dotted line represents the cut-off of immune response. P-values indicating differences in IgG response level at the overall time points (Kruskal-Wallis test) or between two different time points (Wilconxon matched pair test) are presented. Vertical solid grey line indicates timing of VCI.

The effectiveness of the VCI was evaluated by examining changes in IgG Ab response to Nterm-34kDa salivary peptide (Fig 2) and prevalence of “immune responders” (in %, Table 1) before and after the intervention. The median ΔOD value for IgG level decreased significantly until 30 days after VCI (P <0.0001 from T0 to T0+15 and P = 0.0002 from T0+15 to T0+30; Wilcoxon matched paired test). No significant difference was observed between T0+30 and T0+45 time-periods (Fig 2). The proportions of immune responders (Table 1), also decreased from T0 (88.23%) to T0+30 (67.64%), however a slight increase was observed at T0 + 45 (71.56%).

**Table 1:** Entomological parameters in percentage of immune responders before (T0) and after (T0+15; T0+30; T0+45) vector control implementation against Chikungunya transmission in two urban districts of St Denis, La Réunion Island, 2010.

	T0	T0+15	T0+30	T0+45
House index (HI in %)^§^	23.4	6.8	18.5	10.2
Breteau index (BI)^#^	55.5	49.3	55.5	10.2
Pourcentage of immune responders†	82.33%	75.99%	67.64%	71.56%

†: For T0 vs T0+15, **χ**^**2**^ = 5.580, P = 0.01; for T0 vs T0+30 **χ**^**2**^ = 11.15, P = 0.0008; for T0 vs T0+45, **χ**^**2**^ = 8.822, P = 0.003; for T0+15 vs T0+30, **χ**^**2**^ = 1.062, P = 0.3029; for T0+15 vs T0+45, **χ**^**2**^ = 0.4030, P = 0.5256; for T0+30 vs T0+45, **χ**^**2**^ = 0.1596, P = 0.6896

§: House index = Percentage of houses infested with larvae and/or pupae

#: Breteau index = number of positive containers per 100 houses inspected

**Fig 3:**
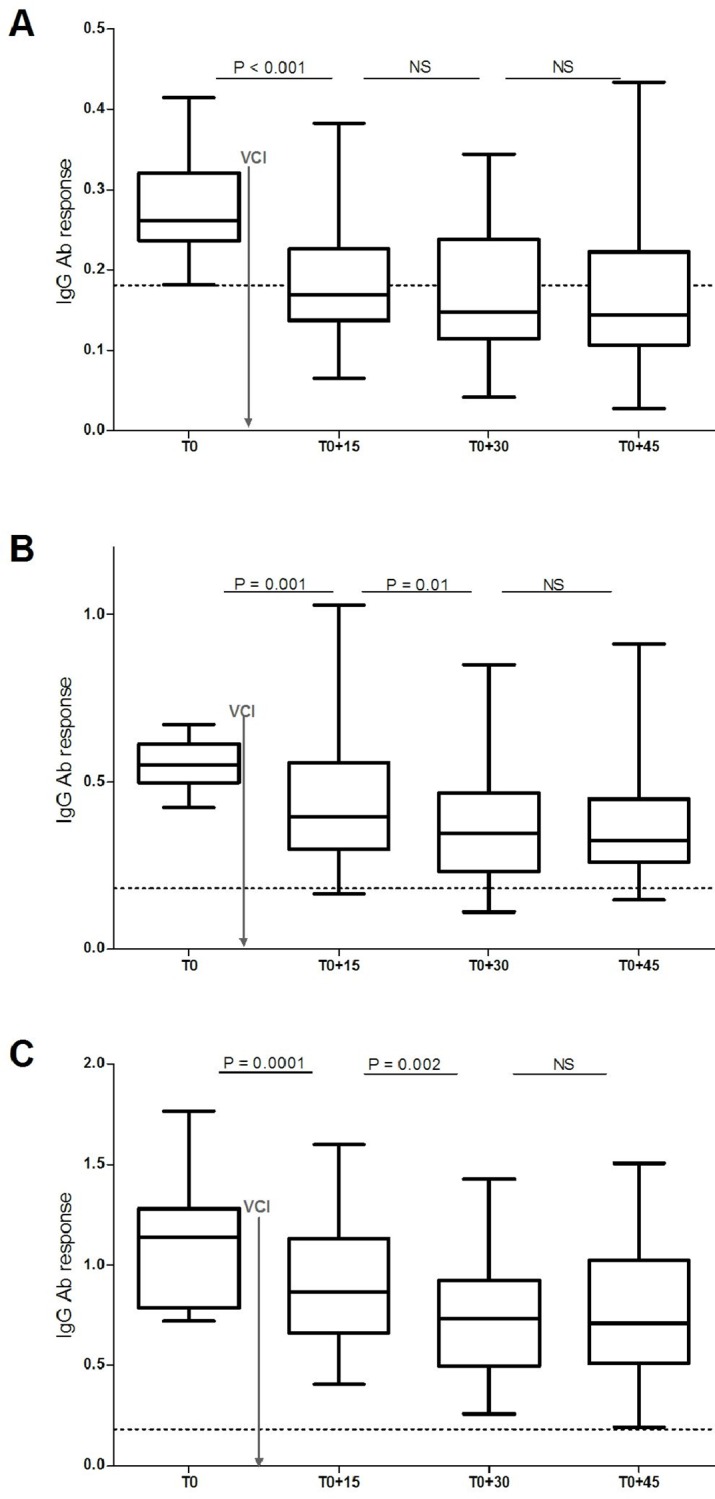
IgG Ab response to Nterm-34kDa salivary peptide from individuals exposed to *Ae*. *albopictus* bites, after vector control implementation and according to the initial level. IgG Ab response before and after vector control was presented for “lower responders” (3A), “medium responders” (3B) and “higher responders” (3C) groups defined according to tertile values of individual ΔOD before vector control (= initial level). Statistical differences of the level of IgG response between two time-points are indicated by P-values estimated by a Wilconxon matched pair test. Vertical solid grey line indicates timing of VCI.

The impact of VCI was also assessed according to the initial level of IgG Ab response in individuals (T0, i.e. before VCI). The “Immune responders” at T0 (i.e: those with ΔOD ≥ 0.181; n = 90) were divided into three groups according to the values of the tercile at T0 (Fig 3): “lower responders” (0.181 ≤ ΔOD ≤ 0.4225; n = 30; Fig 3A), “medium responders” (0.4225 < ΔOD ≤ 0.7301; n = 28; Fig 3B) and “higher responders” (0.7301 < ΔOD≤ 1.765; n = 32; Fig 3C). The changes in IgG Ab response in each group was assessed from T0 to T0+45. For all groups, the IgG level to the Nterm-34kDa peptide decreased progressively. This decrease was significant until T0+30 for “medium” and “higher” groups (Fig 3B and 3C). Interestingly, for the “lower responders” group, the median value of the IgG response was very low from T0+15 and below the cut-off until the end of the follow-up, whereas this point was never observed for the other immunological groups.

Multivariate analysis was performed to assess the influence of potential confounders factors on the IgG Ab response, including: VCI, use of individual protection against mosquito bites, daily commuting out of dwelling house, sex and age. The results presented in Table 2 showed that the level of IgG Ab response to Nterm-34kDa peptide was significantly (P <0.05) influenced by only VCI factor (decrease of the IgG Ab level) whereas, no significant influence of the other factors was observed.

**Table 2:** Multivariate analysis of IgG Ab response to Nterm-34kDa salivary peptide in human after vector control implementation against Chikungunya transmission, in two urban districts of St Denis, La Reunion, 2010.

Effects	Estimate	95% Confidence interval		P-value
Intercept (anti Nterm-34kDa IgG response)	0.5424	0.3718	0.7130	0.0000
Vector Control Intervention^$^	-0.1725	-0.2034	-0.1416	0.0000
Use of individual protection against^£^ mosquito bites	0.0675	-0.0073	0.2085	0.3440
Daily commuting^†^	-0.0252	-0.1643	0.1139	0.7206
Men^¥^	0.0297	-0.1146	0.1741	0.6833
Age	0.0005	- 0.0044	0.0053	0.7206

**$**: compared with IgG Ab response before vector control implementation

**£**: compared with IgG Ab response from individuals using no individual protection

**†**: compared with IgG responses from individuals usually stay at home

**¥**: compared with the level of IgG response from women

### Entomological data and IgG response to Nterm-34kDa salivary peptide

**Fig 4:**
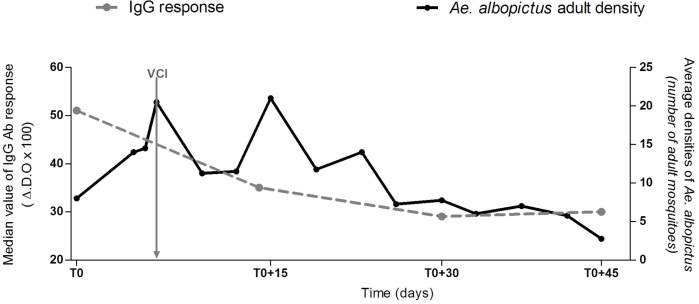
Median of IgG Ab response to Nterm-34kDa salivary peptide from individuals exposed to *Ae*. *albopictus* bites according to adult mosquito density. Evolution of IgG Ab response to Nterm-34kDa salivary peptide (median values) is represented (dotted grey line with circle) with the average density of *Ae*. *albopictus* adult population as estimated every two days during the follow-up (solid black line with circles). The timing of vector control implementation (vertical solid grey line) is represented.

The presence of *Ae*. *albopictus* mosquitoes was estimated through adult mosquito density, BI (Breteau Index) and HI (House Index). The evolution of these entomological parameters during the follow-up are presented in the Table 1 and in the Fig 4. Overall, entomological indicators appeared to decrease during the study. Indeed, except the increase of adult mosquito density observed for a short period at T+15 and the slight increase of HI and BI at T0+30, the values of these parameter remained decreasing until T0+45 after VCI. This decreasing trend appeared to be significant associated to the evolution of the median level of IgG response to Nterm-34kDa salivary peptide (Fig 4) and to the proportion of “immune responders” (Table 1) until the T0+30 time-point. From T0+30 to T0+45, entomological parameters decreased whereas anti-Nterm-34kDa IgG response remained unchanged.

## Discussion

The present study reported, for the first time, the detection of IgG Ab response to *Ae*. *aegypti* Nterm-34kDa salivary peptide in human adult individuals exclusively exposed to *Ae*. *albopictus* bites. It shows that the level of specific Ab response and the proportion of immune responders significantly decreased after VCI. This decrease was detected earlier (2 weeks) and persisted until 4 weeks after VCI. During the first week after VCI, the decrease of the IgG Ab response may be associated to the drop of adult mosquito density. This similar evolution of adult mosquito density and Ab response to the Nterm-34kDa salivary peptide appeared to persist until the end of study. Analysis of the evolution of HI showed a similar pattern with the level of IgG Ab response to the Nterm-34kDa peptide from T0 to T0+30. Firstly these results showed the existence of Ab response to *Ae*. *aegypti* Nterm-34kDa salivary peptide in adult population exposed to *Aedes* mosquito bites, as previously observed in children [32]. In addition and as major point of the present study, these results validate the usefulness of the IgG Ab response to one salivary antigen for evaluating human exposure to *Aedes* bites and for monitoring vector control strategies against arboviral diseases.

Two previous studies had validated IgG Ab response to *Ae*. *aegypti* Nterm-34kDa salivary peptide as pertinent candidate biomarker of *Aedes* bites in African and Asian individuals [32,33]. Here, the IgG Ab response to the Nterm-34kDa peptide was detected in 82.23% at T0 and relevant at least 67.74% (at T0+30) of individuals living in urban area in La Reunion Island where *Ae*. *aegypti* is virtually absent [34] and *Ae*. *albopictus* is highly anthropophilic [37,38]. This highlights cross-reactivity between *Ae*. *aegypti* and *Ae*. *albo*pictus salivary peptides. Such cross-reactivity was already reported in one study describing immunogenic proteins in *Ae*. *albopictus* saliva [39]. Indeed, in this former study, sera from individuals exclusively exposed to *Ae*. *albopictus* or *Ae*. *aegypti* bites, showed similar antigenic profiles by immunoblotting especially for the 34kDa family proteins. In contrast to the high proportion of immune responders observed in our study, another recent study reported only 19% of immune responders for both *Ae*. *aegypti* and *Ae*. *albopictus* SGE [18]. This difference could be probably explained by the fact that the present study specifically targeted Ab response to only one antigen (antigenic peptide) instead of overall antigenic proteins contained in the SGE. It can be hypothesizes that, among this cocktail of proteins, antigenic sequences located of the 34kDa putative protein, are probably low detected by specific Ab in the sera. The present findings highlight the pertinence of the IgG Ab response to *Ae*. *aegypti* Nterm-34kDa salivary peptide to detect human exposure to *Ae*. *albopictus* bites.

The human Ab response to vectors’ salivary proteins is a pertinent tool for evaluating the efficacy of vector control strategies, against malaria [20–22,28,40]. In the present study, a significant decrease of human IgG response to Nterm-34kDa peptide was observed just one week after VCI. As it has been clearly reported an association between the level of human IgG Ab response to salivary proteins and vector densities [15,17,18,40,41], we can hypothesize that the decrease of specific IgG response could be linked to the drop of *Ae*. *albopictus* density and by consequence, to a decrease of human exposure to *Aedes* mosquito bites. The rapid decrease of IgG Ab response to Nterm-34kDa peptide associated to the rapid reduction of entomological indices (adults’ density, HI, BI) emphasized the potential of the IgG response to the Nterm-34kDa peptide to detect rapid variations in human exposure to *Aedes* bites. This indicates that this peptide could be an accurate biomarker for evaluating the short-time efficacy of VCI on human-vector contact.

The early but not long (until 30 days) significant decrease of IgG level to Nterm-34kDa, suggests a rapid but not sustained impact of the VCI done during the present study (combined insecticide treatment and physical elimination of breeding sites), on the density of *Aedes* population. Others studies, reported same quick reduction in entomological parameters after VCI against *Aedes* mosquito [42–45]. It indicated that the IgG response to Nterm-34kDa peptide rapidly dropped after the interruption of human exposure to *Aedes*. Doucoure and his colleagues observed same decrease 6 weeks after VCI, using *Ae*. *albopictus* SGE as antigens [23]. The IgG response to the Nterm-34kDa peptide may be therefore more sensitive than IgG response to SGE for detecting early variations in human exposure to *Aedes* bites. This biomarker property appears appropriate for evaluation of emergency interventions during outbreaks, targeting adult vectors, especially in endemic areas of *Aedes* mosquito.

Related to the three groups of immune responders defined according to the initial level of immune response at T0, specific IgG response rapidly (15 days after VCI) lowered below the cut-off in the “lower immune responders” group. Interestingly, this disappearance of specific IgG response was not observed in “medium” and “higher responders” groups. As previously observed for *Ae*. *albopictus* SGE [23], this result emphasized the short half-life of IgG Ab response to *Aedes* salivary antigen after interruption of human-vector contact, especially in case of low exposure to mosquito bites (i.e. low immune responder at T0). No significant difference of specific IgG level was observed between T0+30 and T0+45 days after VCI. This seems to indicate that vector control strategies implemented in the study area have became ineffective after 30 days. A range of time from 15 to 30 days could be then selected for adequate evaluation of VCI by using such salivary biomarker. Future investigations should precise this timing to improve the operational evaluation of VCI efficacy.

The multivariate analysis showed no influence of sex, age and professional activity in the level of IgG Ab response to Nterm-34kDa after VCI. As previously reported for *Anopheles* salivary biomarker [28], it indicated that this salivary biomarker could be useful whatever sex and age of individuals. However, the exclusion of youngest population (<18 years) in our study represents a significant limitation. This biomarker should be validated for all age groups to demonstrate its full potential.

In conclusion, the results suggest that the Ab response to the *Aedes* Nterm-34kDa peptide represent a relevant tool for evaluating human exposure to *Ae*. *albopictus* vector. This candidate biomarker can detect the short-time variations of human exposure to *Aedes* mosquito bite after vector control implementation. This immuno-epidemiological tool appears relevant to assess the efficacy of vector control against arboviruses vectors.

## Supporting Information

S1 Checklist « Elanga—STROBE Checklist–cohort 1.1 » (DOC)
