# Supplementary material for: Human IgG Antibody Response to Aedes Nterm-34kDa Salivary Peptide, an Epidemiological Tool to Assess Vector Control in Chikungunya and Dengue Transmission Area
Source: PLoS Negl Trop Dis. 2016 Dec 1;10(12):e0005109. doi: 10.1371/journal.pntd.0005109 (PMC5131890; doi:10.1371/journal.pntd.0005109)
Supplement: S1 Checklist — (DOC) [file pntd.0005109.s001.doc]

STROBE Statement—Checklist of items that should be included in reports of ***cohort studies***

|  | Item No | Recommendation |
| --- | --- | --- |
| **Title and abstract** | 1 | (*a*) Indicate the study’s design with a commonly used term in the title or the abstract. [**Within the abstract page 3 (see also methodology section for details of design)]** |
|  |  | (*b*) Provide in the abstract an informative and balanced summary of what was done and what was found.[ **Within results section of the abstract, page 3]** |
| Introduction | | |
| Background/rationale | 2 | Explain the scientific background and rationale for the investigation being reported. [**See the introduction section from line 64 to line 114 (pages 5 – 7)]** |
| Objectives | 3 | State specific objectives, including any prespecified hypotheses.  **[Within the introduction section from line 115 to line 121 (page 7)]** |
| Methods | | |
| Study design | 4 | Present key elements of study design early in the paper. [**This was indicated in the study design and population section (see lines 138 - 155, page 8 )]** |
| Setting | 5 | Describe the setting, locations, and relevant dates, including periods of recruitment, exposure, follow-up, and data collection. [**Within the study site, study design and population sections, in addition see entomological and rainfall data collection section (see lines 131 – 163; pages 7-9)]** |
| Participants | 6 | (*a*) Give the eligibility criteria, and the sources and methods of selection of participants. Describe methods of follow-up. [**This is indicated in study design and population section (see lines 138 – 155, page 8)]** |
|  |  | (*b*) For matched studies, give matching criteria and number of exposed and unexposed.  **[Within study design and population section (see lines 138 – 155, page 8)]** |
| Variables | 7 | Clearly define all outcomes, exposures, predictors, potential confounders, and effect modifiers. Give diagnostic criteria, if applicable. [**This is presented in study design and population section (see lines 138 – 155; page 8) and also in Table 2 for multivariate analysis (page 25). The diagnostic criteria were not applicable**] |
| Data sources/ measurement | 8* | For each variable of interest, give sources of data and details of methods of assessment (measurement). Describe comparability of assessment methods if there is more than one group. [**This is presented in study design and population section (lines 138 – 155; page 8). See also entomological and rainfall data collection section (lines 157-163; page 8-9)]** |
| Bias | 9 | Describe any efforts to address potential sources of bias. [**N/A]** |
| Study size | 10 | Explain how the study size was arrived at. [**Within study site and study design and population sections (lines 131 – 155; page 7-8)]** |
| Quantitative variables | 11 | Explain how quantitative variables were handled in the analyses. If applicable, describe which groupings were chosen and why. [**N/A]** |
| Statistical methods | 12 | (*a*) Describe all statistical methods, including those used to control for confounding. **[This is indicated in the statistical analysis section (lines 188 – 197; page 10)]** |
|  |  | (*b*) Describe any methods used to examine subgroups and interactions.  **[Within Results section (lines 222 – 232; pages 11-12)]** |
|  |  | (*c*) Explain how missing data were addressed. [**N/A]** |
|  |  | (*d*) If applicable, explain how loss to follow-up was addressed. [**N/A]** |
|  |  | (*e*) Describe any sensitivity analyses  **[This is indicated in the statistical analysis section (lines 188 – 197; page 10)]** |
| Results | | |
| Participants | 13* | (a) Report numbers of individuals at each stage of study—eg numbers potentially eligible, examined for eligibility, confirmed eligible, included in the study, completing follow-up, and analysed. **[This is previously presented in study design and population sections (lines 131 – 155; page 7-8)]** |
|  |  | (b) Give reasons for non-participation at each stage. [**N/A]** |
|  |  | (c) Consider use of a flow diagram. [**N/A]** |
| Descriptive data | 14* | (a) Give characteristics of study participants (eg demographic, clinical, social) and information on exposures and potential confounders. **[This is previously presented in study design and population sections (lines 131 – 155; page 7-8)]** |
|  |  | (b) Indicate number of participants with missing data for each variable of interest. [**N/A]** |
|  |  | (c) Summarise follow-up time (eg, average and total amount). **[This is previously presented in study design and population sections (lines 131 – 155; page 7-8)]** |
| Outcome data | 15* | Report numbers of outcome events or summary measures over time.  **[This is previously presented in study design and population sections (lines 131 – 155; page 7-8). Within also in Results section (lines 212-232; pages 11-12)].** |
| Main results | 16 | (*a*) Give unadjusted estimates and, if applicable, confounder-adjusted estimates and their precision (eg, 95% confidence interval). Make clear which confounders were adjusted for and why they were included. [**N/A]** |
|  |  | (*b*) Report category boundaries when continuous variables were categorized. [**N/A]** |
|  |  | (*c*) If relevant, consider translating estimates of relative risk into absolute risk for a meaningful time period. [**N/A]** |
| Other analyses | 17 | Report other analyses done—eg analyses of subgroups and interactions, and sensitivity analyses.  **[This is presented in the statistical analysis section (lines 188 – 197; page 10) and in Results section (Table 2; page 25 and lines 233-238; page 12)].** |
| Discussion | | |
| Key results | 18 | Summarise key results with reference to study objectives.  [**Within start of Discussion section (lines 254 – 267; page 13)]** |
| Limitations | 19 | Discuss limitations of the study, taking into account sources of potential bias or imprecision. Discuss both direction and magnitude of any potential bias.  **[This is indicated in several parts of Discussion section (pages 13-16)]** |
| Interpretation | 20 | Give a cautious overall interpretation of results considering objectives, limitations, multiplicity of analyses, results from similar studies, and other relevant evidence. **[Indicated in several parts of Discussion section (see lines 268 – 332; pages 13-16)]** |
| Generalisability | 21 | Discuss the generalisability (external validity) of the study results  **[Within end of Discussion section (lines 333-343; page 16)]** |
| Other information | | |
| Funding | 22 | Give the source of funding and the role of the funders for the present study and, if applicable, for the original study on which the present article is based. The source of funding is given in the acknowledgements section.  **[See “Financial disclosure” section in submitted PDF]** |

*Give information separately for exposed and unexposed groups.

**Note:** An Explanation and Elaboration article discusses each checklist item and gives methodological background and published examples of transparent reporting. The STROBE checklist is best used in conjunction with this article (freely available on the Web sites of PLoS Medicine at http://www.plosmedicine.org/, Annals of Internal Medicine at http://www.annals.org/, and Epidemiology at http://www.epidem.com/). Information on the STROBE Initiative is available at http://www.strobe-statement.org.
